# Rethinking the Postpartum “Orphan Window” Treatment in Gestational Diabetes Management

**DOI:** 10.3390/jcm15072519

**Published:** 2026-03-26

**Authors:** Angelo Sirico, Lucia Sandullo, Maria Fatigati, Davide Pisani, Giuseppe Maria Maruotti, Luigi Cobellis

**Affiliations:** 1 Department of Woman, Child, and General and Specialized Surgery, University of Campania “Luigi Vanvitelli”, 80138 Naples, Italy; 2 Obstetrics and Gynecology Unit, Department of Public Health, University of Naples Federico II, 80126 Naples, Italy

**Keywords:** Gestational Diabetes Mellitus (GDM), postpartum, type 2 diabetes, prevention, orphan window, myo-inositol

## Abstract

Gestational Diabetes Mellitus (GDM) is the most common metabolic complication of pregnancy, affecting approximately 14% of pregnancies globally. Despite the frequent normalization of glycemic parameters immediately after delivery, GDM is an important precursor of subsequent chronic disease, increasing the risk of type 2 diabetes (T2DM). Current international guidelines suggest just a strictly observational approach during the immediate puerperium, recommending metabolic screening only between 6 and 12 weeks postpartum. This has contributed to the creation of a therapeutic “orphan window” where women receive no specific metabolic support, leaving their metabolic status unassessed and unmanaged. We postulate that the immediate postpartum period represents a critical window of “metabolic plasticity” where the abrupt cessation of placental hormones offers a unique opportunity to restore insulin sensitivity and promote “beta-cell rest” before the onset of irreversible dysfunction. Consequently, this narrative review and perspective examines the epidemiological urgency of the GDM-to-T2DM transition and provides a biological rationale for early pharmacological or nutraceutical intervention. Specifically, we discuss the limitations of metformin and present the hypothesis of myo-inositol combined with alpha-lactalbumin as a safe, lactation-compatible “bridging therapy” to preserve beta-cell function, improve compliance, and modify the natural history of diabetes in this high-risk population, highlighting that this theoretical proposal requires validation through future clinical trials.

## 1. Introduction

Gestational Diabetes Mellitus (GDM) is defined as glucose intolerance with onset or first recognition during the second or third trimester of pregnancy, once overt diabetes prior to pregnancy has been excluded. Maternal overweight and obesity, history of GDM in a previous pregnancy, advanced maternal age, family history of type 2 diabetes mellitus and ethnicity are the main GDM risk factors [1]. GDM is suspected to arise from inadequate maternal β-cell response, with metabolic adaptations mainly in lipid metabolism, worsening the insulin resistance that occurs in healthy pregnant women, who compensate for it through an adapted response of β-cells.

Historically viewed as a transient obstetric complication that resolves with the delivery, evolving epidemiological evidence has fundamentally shifted this paradigm. GDM is now reframed as a chronic condition unmasked by the physiological stress test of pregnancy, signaling a predisposition to future metabolic disease. The relevance of GDM as a risk factor for type 2 diabetes and cardiovascular disease has been widely recognized [2]. According to the International Diabetes Federation (IDF) Diabetes Atlas (2021), hyperglycemia in pregnancy affects approximately 16.7% of live births worldwide, with over 80% of these cases attributable to GDM [3]. While prevalence varies significantly by geography and diagnostic criteria, the trend is universally upward, driven by rising maternal age and the global obesity epidemic.

The clinical resolution of hyperglycemia at delivery is often deceptive. Landmark longitudinal cohorts, including the Hyperglycemia and Adverse Pregnancy Outcome (HAPO) Follow-up Study, have demonstrated that the underlying metabolic defect in GDM survivors is persistent [4]. The relative risk of developing type 2 diabetes (T2DM) is elevated 7- to 10-fold compared to normoglycemic women. The main risk factors for progression to type 2 diabetes are maternal age, family history of type 2 diabetes mellitus, prepartum and postpartum BMI, the necessity of insulin therapy for gestational diabetes, and fasting glucose level during pregnancy [5,6]. Crucially, this conversion is not a distant event of old age; the highest velocity of conversion occurs within the first 3–5 years postpartum. An interesting meta-analysis published in *The Lancet* highlighted that up to 50% of women with GDM will develop overt diabetes within a decade [7].

Despite this ticking metabolic clock, the clinical management of GDM survivors is characterized by a fragmented approach. Once the obstetric risk concludes, the patient is frequently lost in the transition between obstetric and primary care, falling into a zone of therapeutic inertia. In this view, a multisystem approach is very important to retard progression to DM and cardiovascular disease (CVD) in women with GDM [8]. A comprehensive literature search was conducted utilizing the PubMed/MEDLINE and Scopus databases, focusing on articles published within the last decade (up to January 2026). Search terms included ‘Gestational Diabetes’, ‘Postpartum screening’, ‘Type 2 Diabetes prevention’, ‘Myo-inositol’, and ‘Beta-cell rest’. We aimed to synthesize current epidemiological gaps and formulate a biologically sound hypothesis for future clinical trials. 

## 2. The Postpartum Challenge: Between Guidelines and Clinical Reality

A critical review of major international clinical practice guidelines reveals a striking paradox: while there is unanimous consensus on the long-term risk posed by GDM, the immediate postpartum period is treated with a standardized approach of ‘passive waiting’. Evidence-based postnatal care is a fundamental instrument to improving maternal long-term health. However, the global evidence base for postnatal care after GDM needs further improvements [9].

Current international guidelines advise women with a recent history of GDM to immediately stop their blood-glucose lowering therapy and to be screened for T2DM after an observation period ranging from 4 to 12 weeks [10,11,12]. The rationale behind this 6–12-week delay is to allow for the complete involution of pregnancy-related hormonal changes (e.g., placental lactogen clearance) and to avoid immediate postpartum confounders such as blood loss and fluid shifts, which invalidate HbA1c testing. However, this wait creates a critical 4- to 6-week window of ‘beta-cell rest’, the precise duration before residual insulin resistance and lipotoxicity trigger irreversible beta-cell apoptosis. Therefore, leaving this specific period unmanaged is what transforms a physiological recovery phase into a missed therapeutic opportunity Crucially, none of these leading guidelines advocate for a specific pharmacological or nutraceutical intervention during the 0–6 week “orphan window” [13]. Their approach is uniformly reactive. Action is only recommended after dysglycemia is confirmed. The ADA’s Recommendation is an instance, advising that individuals with a history of GDM found to have prediabetes “should receive intensive lifestyle interventions and/or metformin to prevent diabetes”. This recommendation, while vital, is contingent upon a diagnosis made well after the period of maximal metabolic plasticity has passed. The NICE guidelines echo this by identifying the need for further research on “postnatal treatment for women diagnosed with gestational diabetes,” explicitly questioning whether effective long-term pharmacological interventions exist to prevent T2DM onset, thereby acknowledging the current evidence gap that this project aims to fill.

Relying on a 6–12 week OGTT as the sole safety net is currently a failing strategy. Postpartum screening rates remain alarmingly low across diverse healthcare systems globally. Despite international guidelines, real-world compliance is critically insufficient. For instance, data from the U.S. Pregnancy Risk Assessment Monitoring System (PRAMS) showed that only 50.5% of women with GDM received postpartum screening in 2022 [14]. Similar drastic attrition rates have been documented worldwide, ranging from less than 30% in Japan and Canada, to under 50% in European and Chinese retrospective cohorts [15,16,17,18]. This global high attrition rate means that for the vast majority of GDM survivors, the “orphan window” extends indefinitely, leaving their metabolic status completely unmanaged until overt hyperglycemia manifests years later.

The barriers to screening are multifactorial and have been extensively documented in qualitative and quantitative research.

Patient-Level Barriers: Women often report a low perception of long-term risk, a feeling of a “false cure” after delivery, and the overwhelming competing priorities of newborn care, which consistently relegate maternal self-care to a lower priority. The practical burdens of the OGTT itself—the need for fasting, long wait times, and the unpleasantness of the glucose drink—serve as significant deterrents.

HCP and System-Level Barriers: Communication gaps are a major contributor. Qualitative studies from China and Vietnam reveal that women often perceive a lack of interest from healthcare providers (HCPs) regarding their postpartum health, with the clinical focus shifting almost entirely to the infant. Data were obtained from social network platforms and pregnancy groups in China, and they were collected using semi-structured interviews. The attendance rate of postpartum diabetes screening was 35% among those with previous GDM. However, postpartum diabetes screening could be improved through more effective communication from HCPs and increasing the accessibility of screening procedures [19,20]. At a system level, the fragmentation of care between obstetrics, endocrinology, and primary care creates a disjointed care pathway. A recent scoping review by Sushko et al. highlights that while structured diabetes prevention programs (DPPs) are effective at reducing the occurrence of T2D in clinical trials, their real-world application is severely hindered by low recruitment and high attrition rates. Dropout rates are linked to the demands of motherhood and limited access to postpartum care due to lifestyle changes as well as limited resources [21]. However, evidence indicates that DPPs are successful; authors identify the poor transition from prenatal to postpartum care as a primary systemic barrier, reinforcing the notion that the current care model fails to provide a seamless pathway for women during this high-risk period. It is easily understandable that these findings further validate the existence of a ‘therapeutic window’ immediately following delivery, where the lack of integrated, flexible, and early-initiated support may prevent the successful adoption of important lifestyle modifications and a consistent follow-up pathway.

## 3. Metabolic Pathophysiology of GDM Puerperium

Pregnancy induces a state of profound physiological insulin resistance, mediated primarily by placental hormones such as placental lactogen (hPL), cortisol, and progesterone, and, to maintain euglycemia, the maternal pancreas must undergo compensatory hyperplasia and hypertrophy to increase insulin secretion; unfortunately, in GDM cases, this compensation fails due to a pre-existing, often genetically determined, defect in beta-cell function [22]. At delivery, after the expulsion of the placenta, the anti-insulin hormones plummet, leading to a rapid improvement in peripheral insulin sensitivity. Thus, the beta-cells, having been chronically overstimulated in a state of metabolic overdrive, may enter a state of functional exhaustion or, as often defined, “stunning”. The concept of “beta-cell rest”, a phase characterized by the reducing of pancreas secretory demand to allow for cellular recovery, is a well-established principle in T2DM prevention and management. In this view, the immediate postpartum period, with its restored insulin sensitivity, represents an ideal and distinctive window to validate this principle. On the other hand, leaving the pancreas unsupported despite residual insulin resistance and ongoing metabolic stress may accelerate beta-cell apoptosis and de-differentiation, accelerating the progression to T2DM.

The molecular keystones of this postpartum transition are recently beginning to be elucidated. A preclinical study by Bobin et al. [23] used a rodent model of diet-induced GDM to explore perinatal metabolic adaptations in dams with gestational glucose intolerance (IGT) followed by either persistent or resolved postpartum IGT. This study highlighted several key findings: during pregnancy the state of IGT was associated with a distinct metabolic signature characterized by incomplete fatty acid oxidation (FAO), enhanced gluconeogenesis, altered insulin signaling, and systemic oxidative stress. On the other hand, improved glucose tolerance in the postpartum period was linked to the restoration of complete FAO and a significant elevation of nervonic acid-containing sphingomyelins, which have been associated with beta-cell protection. Therefore, the persistence of IGT after delivery was associated with a different set of metabolites known to predict the early onset of insulin and leptin resistance, along with markers of sustained liver dysfunction and muscle insulin resistance. In particular, these findings strongly suggest that specific metabolomic and lipidomic signatures are associated with postpartum GDM outcomes, and the resolution of postpartum IGT appears to rely on specific protective molecular mechanisms, while its persistence would be associated with a multi-organ dysfunction that mirrors the early trajectory toward T2DM. This evidence provides an exceptional convincing biological rationale for targeting these pathways with early interventions.

At a molecular level, GDM is associated with deep alterations in the inositol phosphoglycan (IPG) signaling pathway [1]. In fact, myo-inositol (MI) and D-chiro-inositol (DCI) act as crucial second messengers for insulin signaling [24], and evidence suggests that GDM is characterized by an excessive urinary excretion of inositols and a tissue-specific depletion of intracellular MI, along with the conversion of MI to DCI by the enzyme epimerase, which is an insulin-dependent process. Unfortunately, in states of insulin resistance, this ratio is altered. The inositol resistance impairs the downstream translocation of GLUT4 transporters to the cell membrane, so that even if insulin levels are adequate, glucose uptake is inefficient. This evidence has led researchers to hypothesize that correcting this key signaling deficit immediately in the postpartum period may represent a biologically plausible strategy to restore signaling efficiency and improve glucose homeostasis.

## 4. Redefining the Standard of Care: From Passive Observation to Active Intervention

The practical failure of the postpartum OGTT, due to low adherence, has spurred a search for more accessible and acceptable screening methods after the delivery of women with GDM. While HbA1c and fructosamine are useful in other clinical settings, they lack an acceptable sensitivity and specificity in the immediate postpartum period, largely due to peripartum blood loss and the rapid turnover of red blood cells and proteins, which contribute to reducing their accuracy. Recently, a more promising alternative has been studied with the use of continuous glucose monitoring (CGM) in these patients. A recent study by Elkind-Hirsch et al. [25] evaluated the diagnostic utility of a blinded CGM system in postpartum women with GDM and found that CGM performed comparably to the OGTT in detecting abnormal glucose metabolism, with mean CGM glucose correlating strongly with mean OGTT glucose values. Also, Cabrera et al. [26] found that postpartum CGM was a reasonable and convenient initial screening tool with high completion rates, strong sensitivity, and high acceptability ratings among participants; indeed, data reported that 94% of participants stated that they would prefer CGM over an OGTT. These findings suggest that CGM could serve as a more patient-friendly and effective tool for closing the postpartum screening gap, since the purpose of an active strategy to prevent the progression from GDM to T2DM requires safe, effective, and lactation-compatible agents for this time window. In particular, previous studies set metformin as the gold standard for T2DM prevention, as established by landmark trials like the Diabetes Prevention Program (DPP) [27]. Its efficacy in women with prior GDM is well-documented. However, its use in the immediate postpartum period is controversial for several key reasons.

Metformin is excreted into breast milk and, although the relative infant dose is small and generally considered safe, long-term data on neonatal exposure are limited. Therefore, this lack of long-term safety data creates hesitancy among both clinicians and mothers. However, it is the most extensively studied agent for preventing progression to T2DM in this high-risk population, particularly due to its dual benefit of decreasing insulin resistance and assisting in weight loss [28]. Besides this consideration, metformin use is also often associated with gastrointestinal adverse events, such as diarrhea and nausea, which are poorly tolerated by breastfeeding mothers who require optimal hydration and nutrition for successful lactation. While the Diabetes Prevention Program (DPP) demonstrated the long-term efficacy of metformin in women with prior GDM [29], its initiation during the immediate 0–6 week postpartum period is often delayed due to the frequent screening dropouts and patient hesitancy regarding lactation safety and gastrointestinal tolerability.

Furthermore, from a biological perspective, metformin primarily acts by reducing hepatic glucose production and does not directly target the intrinsic peripheral inositol signaling defects that are characteristic of postpartum GDM women. These are the possible explanations of the reason for considering metformin, a cornerstone of T2DM prevention, not as an optimal agent for the peculiar physiological and practical aspects of the immediate postpartum period.

Currently, nonpharmacological interventions, primarily lifestyle modifications encompassing diet and physical activity, represent a valid alternative of postpartum care [30]. Intensive dietary interventions, such as Very-Low-Calorie Diets (VLCDs), have been shown to facilitate postpartum weight loss and improve insulin sensitivity, although their safety and feasibility in lactating mothers require extreme caution [31]. However, translating these benefits into real-world clinical practice is highly challenging. Qualitative studies highlight significant barriers to lifestyle modifications in the immediate postpartum period. Women frequently report a lack of knowledge regarding future diabetes risk, overwhelming fatigue, and the prioritization of newborn care over personal health, leading to suboptimal dietary and physical activity behaviors [32,33]. To overcome these barriers, structured follow-up programs and innovative digital health solutions have been investigated. For instance, digital T2DM prevention applications (such as the Baby Steps program) have shown promise in supporting behavior change and self-management by providing accessible, tailored education to multi-ethnic cohorts [34]. Nevertheless, real-world data from specialized practices continue to show that engagement and retention in these programs remain suboptimal, highlighting the persistent systemic gap between obstetric discharge and metabolic follow-up [35].

**Figure 1:**
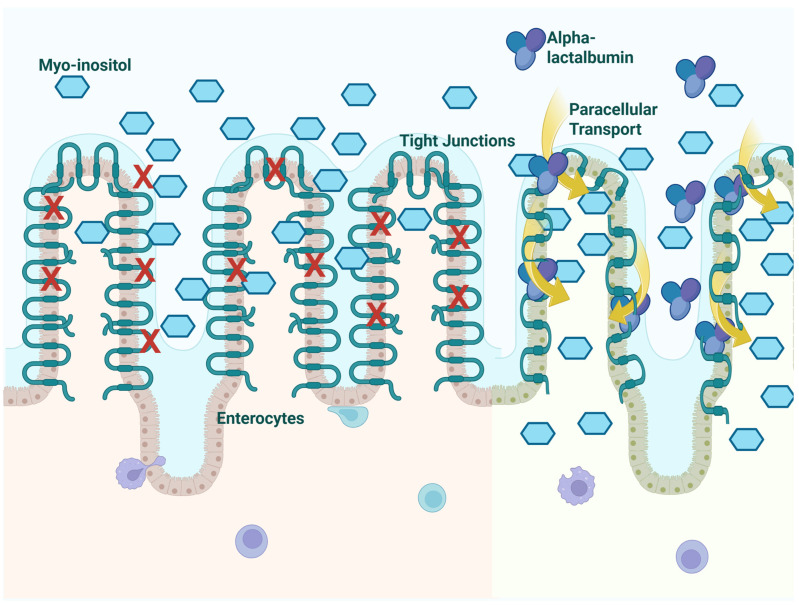
Proposed synergistic mechanism of action of the myo-inositol (MI) and α-Lactalbumin (ALA) combination. In conditions of chronic low-grade inflammation typical of GDM and the postpartum period, the intestinal absorption of inositols may be impaired due to altered tight junction permeability and downregulated transporters. ALA peptides act on intestinal tight junctions, transiently increasing permeability and enhancing the paracellular bioavailability of orally administered MI. Once into the bloodstream, MI could reach target tissues (skeletal muscle and the liver), where it acts as a crucial second messenger for insulin signaling, facilitating PI3K/Akt pathway activation and GLUT4 translocation to the cell membrane, thereby improving glucose uptake.

Myo-inositol (MI) is a carbocyclic sugar alcohol, classified as an insulin sensitizer, that directly targets a core pathophysiological defect in GDM. The evidence supporting its use is growing, as a review by D’Anna et al. confirmed that antenatal MI supplementation reduces the incidence of GDM [24], and other studies also showed that MI supplementation may improve insulin resistance in patients already diagnosed with GDM. While large-scale postpartum trials are lacking, small-scale studies have shown promising improvements in HOMA-IR and fasting insulin levels compared to diet alone. As a natural and vital component of breast milk essential for neonatal development, MI supplementation is considered safe during lactation, and it is generally well-tolerated with no significant GI side effects, offering a distinct advantage over metformin for the postpartum population. On the other hand, a known limitation of MI monotherapy is the existence of “non-responders” (approximately 30–40% of patients), which is thought to be due to poor intestinal absorption or gut dysbiosis. In this view, alpha-lactalbumin (ALA), a whey protein, has been shown to enhance the bioavailability of MI. A study by Monastra et al. [36] demonstrated that ALA modulates the permeability of intestinal tight junctions, facilitating the passive transport of inositols. The combination of MI + ALA, therefore, is hypothesized to represent an optimized formulation that might ensure adequate therapeutic levels are reached even in patients with compromised gut absorption (Figure 1).

Based on this evidence, a paradigm shift from passive observation to proactive support could be necessary, even if this requires the development and rigorous testing of a new therapeutic concept, which we could define as a “bridging protocol”. This protocol should be conceived as a short-term, safe, and well-tolerated intervention initiated immediately postpartum, and its primary goal should not be a long-term treatment but should only be used to provide crucial metabolic support during the vulnerable transition phase of this “orphan window” until the postpartum oral glucose tolerance test (OGTT). Currently, this therapeutic void lacks any established pharmacological or nutraceutical protocols and proposal for interventions that can fill this gap are necessary.

While several pathways could be targeted, we believe that a particularly rational and mechanistically targeted approach may involve studying the combination of myo-inositol (MI) and alpha-lactalbumin (ALA). Indeed, this combination may be particularly adequate for this purpose, since it directly addresses the underlying inositol signaling defect central to GDM pathophysiology and presents an excellent safety profile highly compatible with lactation. Moreover, the inclusion of ALA could maximize MI bioavailability, potentially overcoming the known “non-responder” issue of MI monotherapy.

While lifestyle modifications and structured programs (with or without metformin) must remain the ultimate long-term goal for T2DM prevention, they often fail to be implemented during the very first weeks postpartum. It is precisely within this vulnerable implementation gap that our proposed MI+ALA protocol is intended to act, offering an immediate, well-tolerated, and passive metabolic support while the mother recovers and prepares for long-term lifestyle changes. To translate this biological rationale into a tangible clinical protocol, we propose a supplementation regimen based on established antenatal safety profiles. In particular, a logical starting point for investigation would be a daily dose of 4000 mg of myo-inositol combined with 50 mg of alpha-lactalbumin, divided into two administrations, since this specific dosage is designed to ensure therapeutic circulating levels while maintaining optimal gastrointestinal tolerability during the puerperium.

Future clinical trials should therefore be designed to evaluate whether a six-week course of such a nutraceutical combination can significantly reduce the incidence of dysglycemia (impaired fasting glucose, impaired glucose tolerance, or T2DM) at the 6-week postpartum OGTT. This intervention would have also side benefits. By providing a tangible intervention in this time window, it keeps the patient actively engaged in her own health immediately after delivery. This may act as a psychological and behavioral bridge, reinforcing the importance of the T2DM prevention, improving adherence to the critical 6-week clinical assessment, and fostering a smoother transition into long-term follow-up and preventive care.

**Figure 2:**
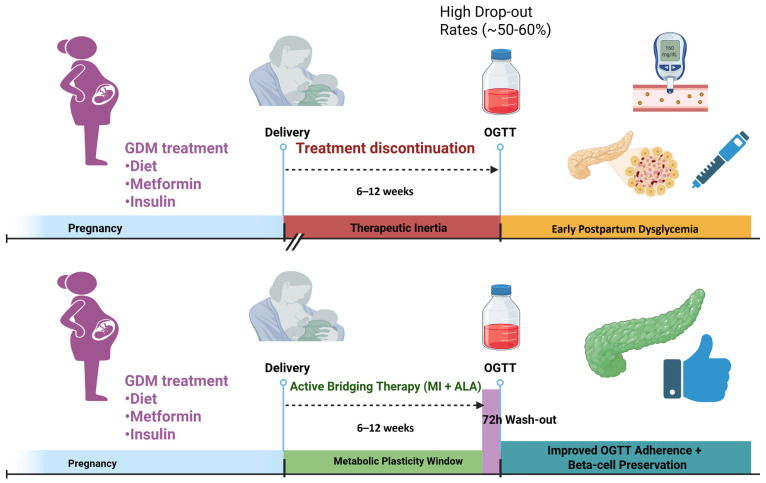
Schematic representation of the proposed “bridging protocol” versus current standard of care. The timeline illustrates the immediate postpartum period (the “orphan window”) in GDM pregnancies. Current guidelines (upper red area) involve treatment discontinuation and passive observation until the scheduled oral glucose tolerance test (OGTT) at 6–12 weeks. The proposed active intervention (bottom green area) initiates myo-inositol and α-lactalbumin supplementation immediately after delivery.

To scientifically validate this proactive approach, future RCTs must distinguish between the acute, transient pharmacological effect of MI (symptomatic relief) and a true structural improvement in beta-cell health (disease modification/legacy effect). Therefore, we strongly propose that the standard 6-week OGTT should be performed only after a 72 h ‘wash-out’ period from the MI+ALA intervention. Given the short pharmacokinetic half-life of MI, a 3-day suspension ensures the complete elimination of exogenous inositols. Furthermore, regarding the ethical implications and feasibility of this design, temporarily halting a nutraceutical supplement for 3 days poses no ethical risk to the patient, especially considering that the current standard of care offers zero metabolic treatment during this period. If patients treated with MI+ALA demonstrate superior OGTT results after this wash-out compared to a placebo group, it would provide the first solid evidence of structurally preserved beta-cell function, rather than a mere masking of underlying dysglycemia (Figure 2).

## 5. Conclusions

The transition from GDM to T2DM is not a uniform process but a complex journey influenced by a broad heterogeneity, and the traditional view of GDM as a single entity is being recently challenged by a more nuanced understanding of its different etiologies. Recent research has begun to subtype GDM based on the dominant metabolic defect identified during the diagnostic antepartum OGTT. A cohort study by Van et al. [37] on 1005 women from the SWIFT study identified three distinct subtypes: isolated postload glucose intolerance (GD-P), fasting hyperglycemia defects (GD-F), and mixed defects (GD-M). These subtypes exhibited statistically significant, graded increases in the risk of postpartum prediabetes, with GD-P having the most favorable profile, GD-F being intermediate, and GD-M being the most predisposed. This heterogeneity powerfully argues against a one-size-fits-all “wait-and-see” strategy. The efficacy of the proposed MI+ALA intervention may not be uniform across these subtypes. Women with fasting (GD-F) or mixed defects (GD-M), which are primarily characterized by profound peripheral insulin resistance, stand to benefit the most from the insulin-sensitizing properties of myo-inositol. Conversely, those with isolated postload defects (GD-P), driven more by insulin secretion failure, might show a different response rate. Future trials must adopt a personalized medicine approach, stratifying outcomes based on these antepartum diagnostic phenotypes. In this view, our proposal of a bridging protocol aligns not only with the biological necessity for early metabolic support but also with the principles of equitable, patient-centered care.

However, it is crucial to emphasize that the proposed ‘bridging protocol’ currently remains a theoretical framework. While it is built upon a strong and biologically plausible pathophysiological rationale, the direct clinical evidence supporting the use of MI+ALA in the immediate 0–6 week postpartum window is still limited. Therefore, this concept must be clearly distinguished from established evidence-based approaches. The implementation of this bridging therapy in routine clinical practice cannot be recommended at this stage and strictly requires prior validation through well-designed, large-scale randomized controlled trials (RCTs) assessing both safety and long-term metabolic efficacy.

A recent global consensus implementation, involving clinicians and women with lived experience from five continents, identified “universal access,” “evidence-based,” and “equity-driven” as the core values and principles for implementing T2DM prevention after GDM [38]. A simple, affordable, and safe nutraceutical intervention like MI+ALA may represent a scalable strategy, particularly in low-resource settings or for underserved populations who face the greatest barriers to accessing postpartum care. The postpartum period for women with a history of GDM is not a period of metabolic silence but a critical and dynamic intersection where the trajectory of a woman’s future health is determined, and in this view the initial postpartum period represents a significant and actionable gap in the continuum of care, contributing to high rates of missed screening opportunities and unmanaged, progressive metabolic dysfunction. Proactive intervention during this period of heightened “metabolic plasticity” is a logical, biologically sound, and (it seems to be) ethical imperative to reduce the burden of T2DM progression, which impacts the life of women and their families and has an increasing cost for national health systems.

## Data Availability

No new data were created or analyzed in this study.

## Abbreviations

The following abbreviations are used in this manuscript:

ADA	American Diabetes Association
ALA	α-Lactalbumin
BMI	Body Mass Index
CGM	Continuous Glucose Monitoring
CVD	Cardiovascular Disease
DCI	D-chiro-inositol
DPP	Diabetes Prevention Program
FAO	Fatty Acid Oxidation
GDM	Gestational Diabetes Mellitus
GLUT4	Glucose Transporter type 4
HAPO	Hyperglycemia and Adverse Pregnancy Outcome
HbA1c	Glycated Hemoglobin
HCP	Healthcare Provider
HOMA-IR	Homeostatic Model Assessment for Insulin Resistance
hPL	Human Placental Lactogen
IDF	International Diabetes Federation
IFG	Impaired Fasting Glucose
IGT	Impaired Glucose Tolerance
IPG	Inositol Phosphoglycan
MI	Myo-inositol
NICE	National Institute for Health and Care Excellence
OGTT	Oral Glucose Tolerance Test
T2DM	Type 2 Diabetes Mellitus
WHO	World Health Organization
